# Effect of Integrated Fertilizer Management on Seed Oil Content, Protein and Fatty Acid Composition of Sunflower Under Rainfed Conditions in Hungary

**DOI:** 10.3390/plants15172602

**Published:** 2026-08-26

**Authors:** Asma Haj Sghaier, Ákos Tarnawa, Hussein Khaeim, András Varga, Kiet Anh Huynh, Noriza Binti Khalid, Viola Kunos, Zoltán Kende

**Affiliations:** 1 Institute of Agronomy, Hungarian University of Agriculture and Life Sciences, Páter Károly u. 1, 2100 Gödöllő, Hungary; 2 Field Crops Department, College of Agriculture, University of Al-Qadisiyah, Al Diwaniyah 58002, Iraq; 3 Doctoral School of Agricultural and Food Sciences, Hungarian University of Agriculture and Life Sciences, Páter Károly u. 1, 2100 Gödöllő, Hungary

**Keywords:** oil content, crude protein, fatty acid profile, oleic acid, linoleic acid, effective microorganisms, potassium, integrated nutrient management

## Abstract

Integrated nutrient management reduces reliance on chemical fertilizers by combining organic and inorganic inputs. A field experiment was conducted under rainfed conditions in Hungary from 2022 to 2024 to evaluate organic, inorganic and biological fertilizers applied to the high-oleic sunflower hybrid ES Emeric. Seven treatments were compared, namely, an unfertilized control, potassium (K), combined organic and inorganic nitrogen (GOIM), effective microorganisms (EM-1), and the combinations K+GOIM, K+EM-1 and GOIM+EM-1. Seed oil, crude protein and moisture content were determined, together with the fatty acid profile of the oil. Growing season influenced every measured variable far more strongly than fertilization, and all treatment responses were expressed as year-by-treatment interactions. Mean oleic acid content was 69.9% in the dry season of 2022 and 85.3% in 2024, but only 28.9% in the cooler and wetter season of 2023, when linoleic acid reached 59.8%. In 2022, K and K+EM-1 gave the numerically highest oil contents, 48.7% and 48.0%, less than one percentage point above the control, while GOIM+EM-1, GOIM and K+GOIM gave significantly higher protein contents than the remaining treatments. In the same season, EM-1 and K+GOIM raised linoleic and alpha-linolenic acids and, therefore, total polyunsaturated fatty acids, whereas GOIM+EM-1 and K increased oleic acid and total monounsaturated fatty acids. Integrated fertilization can therefore be used to shift the balance between monounsaturated and polyunsaturated fatty acids in sunflower oil, but the size and direction of the shift are governed by the conditions of the growing season.

## 1. Introduction

Sunflower (*Helianthus annuus* L.) is one of the most important oilseed crops worldwide, ranking fourth in economic value after soybean, oil palm, and canola [1,2]. Native to North America, sunflower was introduced to Europe during the sixteenth century [3,4]. At present, sunflower is cultivated on approximately 28 million hectares globally and contributes nearly 8% of total world oilseed production [5]. Owing to its wide ecological adaptability, sunflower can be grown under diverse environmental conditions, including drought-prone and semi-arid regions, and on a wide range of soil types, making it a strategically important crop for global oilseed supply [6]. In Europe, and particularly in Hungary, sunflower plays a major role in agricultural production due to its ability to thrive under warm summer conditions and well-drained soils [7]. Hungary ranks among the leading sunflower-producing countries, with approximately 679,600 ha under cultivation and an annual production of about 1.7 million tons [3,4,8]. These characteristics make sunflower cultivation increasingly important under the constraints imposed by climate change. Sunflower seeds are valued for their high oil and protein contents. Sunflower oil is widely used for human consumption and for industrial applications, such as biodiesel, lubricants and oleochemical products, and its market value is closely linked to crude oil prices [7,9]. The oil is rich in unsaturated fatty acids, particularly oleic and linoleic acids, which are associated with beneficial effects on human cardiovascular health [10]. Although sunflower is considered more drought-tolerant than many other crops, its productivity remains highly sensitive to environmental stress, especially drought and high temperatures occurring during critical growth stages from germination to seed filling [11]. Fertilization is a key agronomic practice for improving sunflower seed quality and oil composition. Nutrient supply during seed filling governs how assimilates are partitioned between lipid and protein synthesis, and it also influences the activity of the desaturase enzymes that determine the ratio of oleic to linoleic acid in the oil. Balanced fertilization, particularly with nitrogen and potassium, is therefore crucial for improving crop performance and enhancing tolerance to drought stress [12]. Organic fertilizers such as manure and compost improve soil physical properties by increasing organic matter content and enhancing soil water-holding capacity [13]. Consequently, optimal sunflower performance depends on both sufficient water availability and appropriate nutrient management throughout the crop growth cycle. In this context, integrated nutrient management (INM), defined as the combined use of organic, inorganic, and biological inputs, has been widely promoted as a sustainable strategy to improve soil fertility, reduce dependence on mineral fertilizers, and increase crop productivity. Several studies have demonstrated that combining farmyard manure (FYM) or compost with reduced rates of mineral fertilizers can significantly enhance sunflower growth, physiological traits, and yield. Dambale et al. [14] reported that application of the recommended dose of fertilizers together with 5 t ha^−1^ of FYM increased plant height, leaf area index, and total dry matter accumulation. Similarly, Janmohammadi et al. [15] observed a 17% increase in achene yield with 15 t ha^−1^ of FYM, although the effect was lower than that obtained with full NPK fertilization. The type and source of organic manure also strongly influence crop response. Buriro et al. [16] showed that poultry manure applied at 6–8 t ha^−1^ in combination with 50–75% of NPK improved plant structure, seed yield, and oil content, while goat and sheep manure were more effective than buffalo manure as partial substitutes for mineral fertilizers. Other studies have emphasized the benefits of integrating organic materials with mineral nutrients under stress conditions. Khodaei-Joghan et al. [17] reported that combining bovine manure with compost or zeolite improved growth, seed yield, and water-use efficiency. Vermicompost-based strategies have also been widely investigated. Kumar et al. [18] documented increases in dry matter accumulation and seed traits when compost or vermicompost was incorporated into recommended fertilization programs. Elankavi [19] demonstrated that combining vermicompost with foliar-applied micronutrients significantly enhanced leaf area index, dry matter, and 100-seed weight. Ramesh [20] reported that pressmud-based vermicompost supplying 75% of nitrogen increased oil yield, indicating that vermicompost can partially substitute chemical nitrogen fertilizers. Sefaoğlu [21] similarly confirmed that integrating vermicompost with mineral fertilizers improved oil content and overall agronomic performance.

Moreover, microbial inoculants and biofertilizers play an increasingly important role in nutrient cycling and crop productivity. The combined use of FYM with *Trichoderma viride* [22], *Azotobacter* and phosphate-solubilizing bacteria [23,24], and sulfur-oxidizing bacteria [25] significantly enhanced biomass production, nutrient availability, and both seed and oil yields. Effective microorganisms (EM-1), a consortium of beneficial microbes, have produced promising results in improving yield and quality in wheat [26] and represent a potentially low-cost and environmentally friendly biological input for sunflower cultivation. Potassium, although often receiving less attention than nitrogen and phosphorus in sunflower nutrition, plays a crucial role in enzyme activation, osmotic regulation, stress tolerance, and oil synthesis. Sayed et al. [27] demonstrated that foliar application of 3% KNO_3_ in combination with 14 t ha^−1^ poultry manure significantly improved sunflower yield under drought conditions. Waghmare et al. [28] reported that combining full NPK fertilization with FYM and micronutrients such as zinc and iron resulted in marked increases in head diameter, seed number, and oil yield. Popoola et al. [29] further emphasized the importance of integrating macro- and micronutrients with organic amendments for comprehensive improvements in sunflower growth and productivity. Despite this body of work, three gaps remain. First, the available evidence on integrated nutrient management in sunflower has been obtained almost exclusively on standard linoleic genotypes. Akbari et al. [30], for example, combined farmyard manure, mineral nitrogen and the biofertilizers *Azospirillum* and *Azotobacter* in a single irrigated season and reported oleic acid contents of 37.3% to 40.7% and linoleic acid contents of 48.1% to 53.3% in the cultivar Alestar, a range characteristic of the linoleic type. Whether comparable fertilizer combinations act in the same way in a high-oleic hybrid, in which the oleic acid content is largely fixed by a mutated desaturase allele, has not been established. Second, potassium is rarely included as an experimental factor in such studies, and its combination with effective microorganisms has not been examined, although the physiological role of potassium and the biological benefits of EM-1 have each been documented separately. Third, integrated nutrient management trials on sunflower oil quality are usually confined to a single season, so it is not known whether an effect on the fatty acid profile persists when the same treatments are repeated in seasons that differ in temperature and rainfall. The present study addresses these three gaps under rainfed conditions. It was hypothesized that the integrated application of organic nitrogen from cattle manure, inorganic nitrogen, potassium and effective microorganisms would modify seed oil and protein accumulation and shift the fatty acid composition of the oil relative to non-integrated or single-source fertilization strategies. It was further expected that the size and direction of these responses would depend on the temperature and rainfall conditions of the growing season.

The objectives of this study were threefold. The first was to determine the effect of organic nitrogen, inorganic nitrogen, potassium and effective microorganisms on seed oil, crude protein and moisture content across three consecutive growing seasons. The second was to quantify the resulting changes in the fatty acid composition of the oil and in the derived indices of oil stability. The third was to assess how stable these responses were across seasons that differed markedly in temperature and rainfall.

## 2. Results

### 2.1. Effect of Fertilizer Treatment and Year on Oil and Protein Content

Two-way multivariate analysis of variance showed that the seed quality parameters differed significantly according to both fertilizer treatment and growing season. The multivariate test was significant for fertilizer treatment (Wilks’ lambda = 0.35, F(18, 113.62) = 2.84, *p* < 0.001), for year (Wilks’ lambda = 0.01, F(6, 80) = 117.04, *p* < 0.001) and for their interaction (Wilks’ lambda = 0.03, F(36, 118.91) = 7.41, *p* < 0.001). For oil content, the univariate analysis showed no significant main effect of fertilizer treatment (F(6, 42) = 1.26, *p* = 0.29), a significant main effect of year (F(2, 42) = 33.52, *p* < 0.001) and a significant interaction between year and fertilizer treatment (F(12, 42) = 6.53, *p* < 0.001). Oil content is therefore discussed below as a within-season response rather than as a general treatment effect.

**Figure 1:**
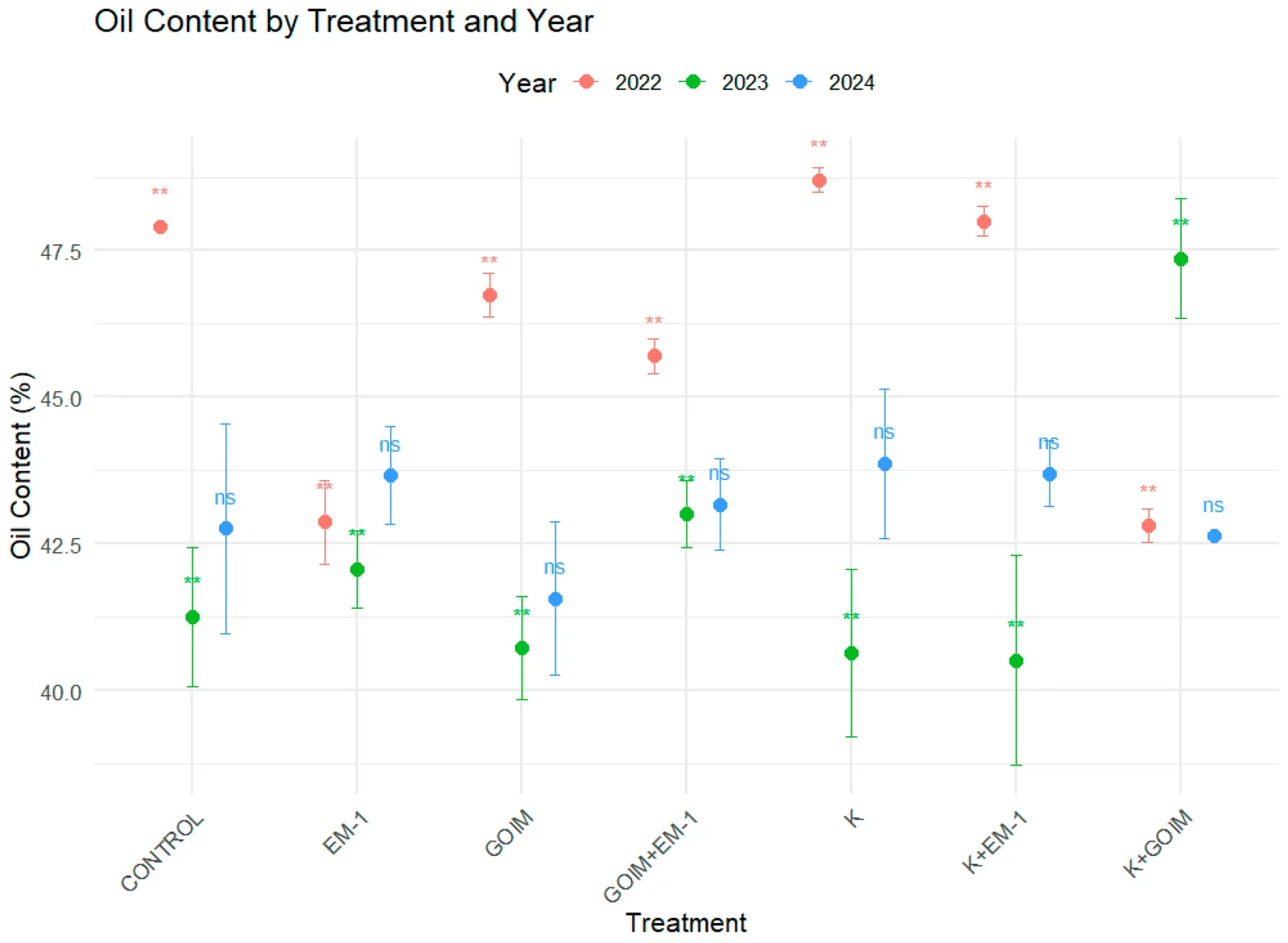
Oil content (%) of sunflower seeds under different fertilizer treatments in 2022, 2023, and 2024. Data are presented as mean ± SD (*n* = 3). Colored symbols represent the three experimental years. Error bars indicate the standard deviation. Asterisks denote the significance of the year × treatment interaction: ns, not significant (*p* ≥ 0.05); * *p* < 0.05; ** *p* < 0.01; *** *p* < 0.001.

Averaged across treatments, oil content was highest in 2022 at 46.1%, compared with 42.3% in 2023 and 43.0% in 2024. A significant difference between years was found for K+GOIM between 2022 and 2023, and for K+EM-1 between 2023 and 2024. In 2022, the numerically highest oil contents were produced by K at 48.7% and K+EM-1 at 48.0%, followed by the control at 47.9%, whereas EM-1 and K+GOIM remained near 42.8%. The three highest values therefore differed by less than one percentage point, and the ranking among them should not be read as a treatment effect. In 2023, the ranking of the treatments changed, with K+GOIM reaching the highest oil content at 47.3% and GOIM+EM-1 the second highest at 43.0%, while the remaining treatments stayed between 40.5% and 42.7%. In 2024, the treatment means were close to one another, ranging from 41.6% under GOIM to 43.9% under K. This reversal of the treatment ranking between 2022 and 2023 is the origin of the significant interaction between year and fertilizer treatment (Figure 1).

**Figure 2:**
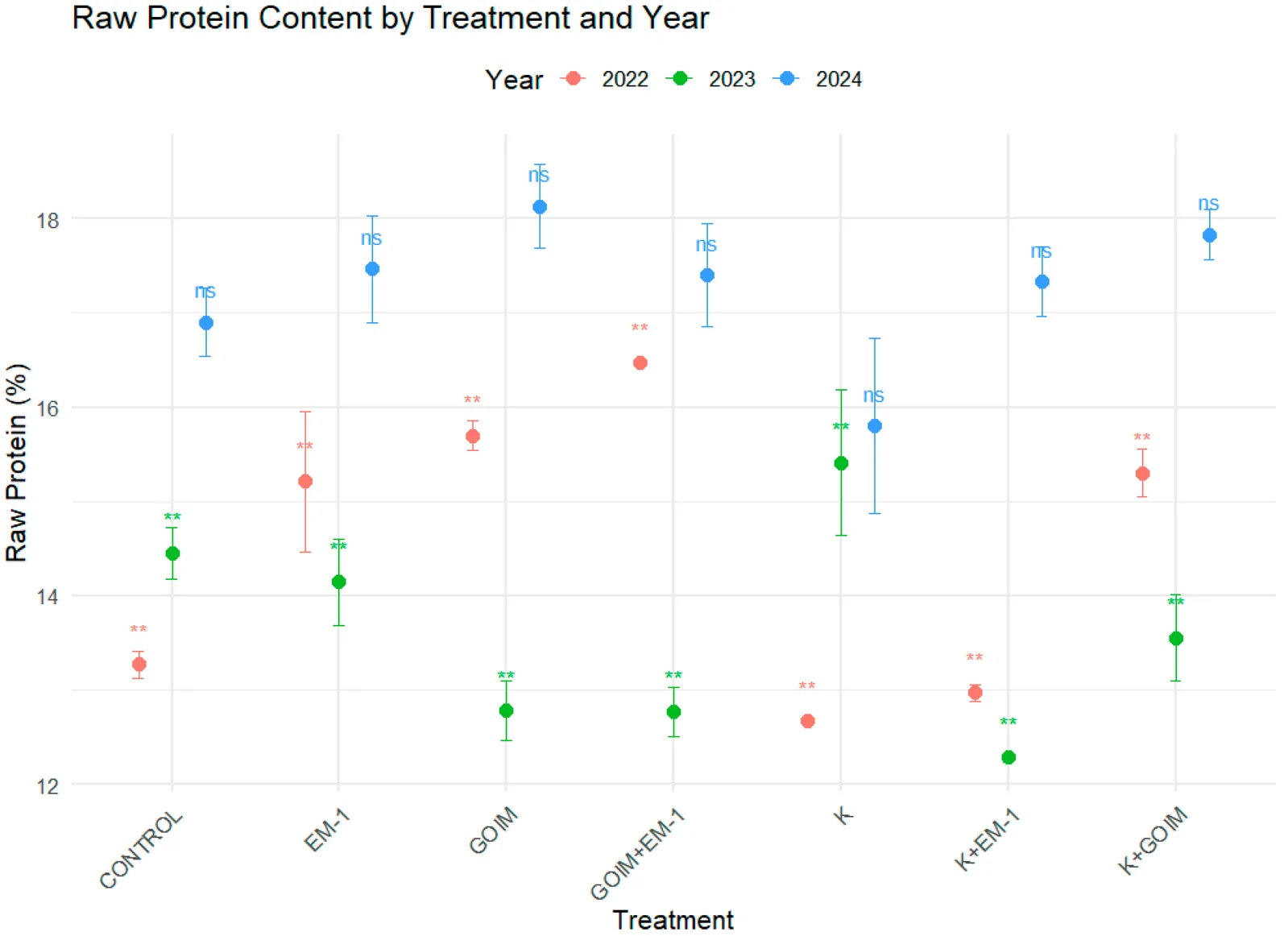
Effect of fertilizer treatments on protein content (%) in 2022, 2023, and 2024. Data are presented as mean ± SD (*n* = 3). Colored symbols represent the three experimental years, and error bars indicate the standard deviation. Asterisks denote the significance of the year × fertilizer treatment interaction: ns, not significant (*p* ≥ 0.05); * *p* < 0.05; ** *p* < 0.01; *** *p* < 0.001.

A notable main effect of year (*p* < 0.001), a substantial effect of fertilizer treatment (*p* < 0.001), and a significant interaction between year and fertilizer treatment (*p* < 0.001) on protein levels were identified using a multivariate two-way ANOVA. The raw protein content of sunflower seeds significantly affects their nutritional value. Figure 2 highlights the protein response influenced by fertilizer treatment and year factors. Averaged across treatments, protein content was 14.5% in 2022, 13.6% in 2023 and 17.4% in 2024. Between 2022 and 2023, there was a considerable reduction in protein levels for GOIM, EM-1, GOIM+EM-1, and K+EM-1. The other treatments did not show any significant differences. In 2022, the highest protein contents were recorded for GOIM+EM-1 at 16.5%, GOIM at 15.7%, K+GOIM at 15.3% and EM-1 at 15.2%. Conversely, the protein levels were significantly lower in the K, K+EM-1, and control treatments. In 2023, the K treatment reached the highest protein content at 15.43%, which was significantly higher than that of the other treatments. In 2024, the protein content was higher in every treatment than in the two preceding seasons and ranged from 15.8% under K to 18.1% under GOIM and K+GOIM, with no significant difference between treatments. GOIM+EM-1 therefore gave the highest protein content in 2022 and K in 2023, whereas in 2024, the highest values were obtained under GOIM and K+GOIM, and K gave the lowest, which illustrates the significant interaction between year and fertilizer treatment.

### 2.2. Effect of Fertilizer Treatment and Year on Fatty Acid Composition

The chromatographic chemical analysis of sunflower oil facilitated the identification of various fatty acids, ranging from C14 to C24, as shown in the table. Based on the statistical analysis, the composition of fatty acids in sunflower oil was notably influenced by both year and the type of fertilizer applied, as well as the interaction between these two factors. The multivariate test was significant for fertilizer treatment (Wilks’ lambda < 0.01, F(108, 150.45) = 2.72, *p* < 0.001), for year (Wilks’ lambda < 0.01, F(36, 50) = 117.59, *p* < 0.001) and for the interaction between fertilizer treatment and year (Wilks’ lambda < 0.01, F(216, 279.46) = 2.09, *p* < 0.001).

#### 2.2.1. Monounsaturated Fatty Acids

Oleic acid (C18:1), a monounsaturated fatty acid, significantly influences the quality of sunflower oil. In the present study, its concentration ranged from 28.2% to 87.2% across the three seasons (Table 1). The level of oleic acid was notably affected by treatment (*p* < 0.001), year (*p* < 0.001), and the interaction between year and fertilizer treatment (*p* < 0.001). The results obtained from the multivariate two-way ANOVA and post hoc pairwise comparisons, conducted using Games–Howell’s method, are shown in Table 1. Averaged across treatments, oleic acid content was 69.9% in 2022 and 85.3% in 2024, compared with 28.9% in 2023. Significant effects of fertilization treatments were observed in 2022 and 2024. In 2022, the highest oleic acid concentrations were recorded with the treatments GOIM+EM-1 (79.65%) and K (76.25%). In 2023, the treatment means were confined to a narrow range, from 28.2% under K to 30.4% under GOIM, and did not differ significantly. In 2024, the highest oleic acid contents were recorded for EM-1 at 87.2%, K+GOIM at 86.8% and GOIM+EM-1 at 86.2%, but no significant difference between treatments was found in that year (Games–Howell, *p* > 0.05). In sunflower oil, eicosenoic acid (C20:1) is another monounsaturated fatty acid found in lower amounts compared to oleic acid (0.14–0.28%). The concentration of eicosenoic acid varied between 2022 and 2024. This acid showed a significant year effect (*p* < 0.001) (Table 1). Between 2022 and 2024, the eicosenoic acid content rose markedly under EM-1, from 0.207% to 0.273%, and under K+GOIM, from 0.208% to 0.277%, corresponding to increases of 32% and 33%. In the control, GOIM and GOIM+EM-1, the change did not exceed 5%, while under K and K+EM-1, the content decreased slightly, by 2.5% and 3.1%. This fatty acid revealed a significant interaction effect between year and treatment (*p* = 0.04). In 2022, the oil from the K treatment had the numerically highest eicosenoic value (0.28%), although the main effect of fertilizer treatment on this fatty acid was not significant (*p* = 0.16) (Table 1). In summary, in 2022, the K treatment produced the highest contents of both eicosenoic and oleic acids, which are the main monounsaturated fatty acids. In 2022, GOIM+EM-1 yielded the highest content of oleic acid, followed, in decreasing order, by the K and K+EM-1 treatments.

#### 2.2.2. Saturated Fatty Acids

**Figure 3:**
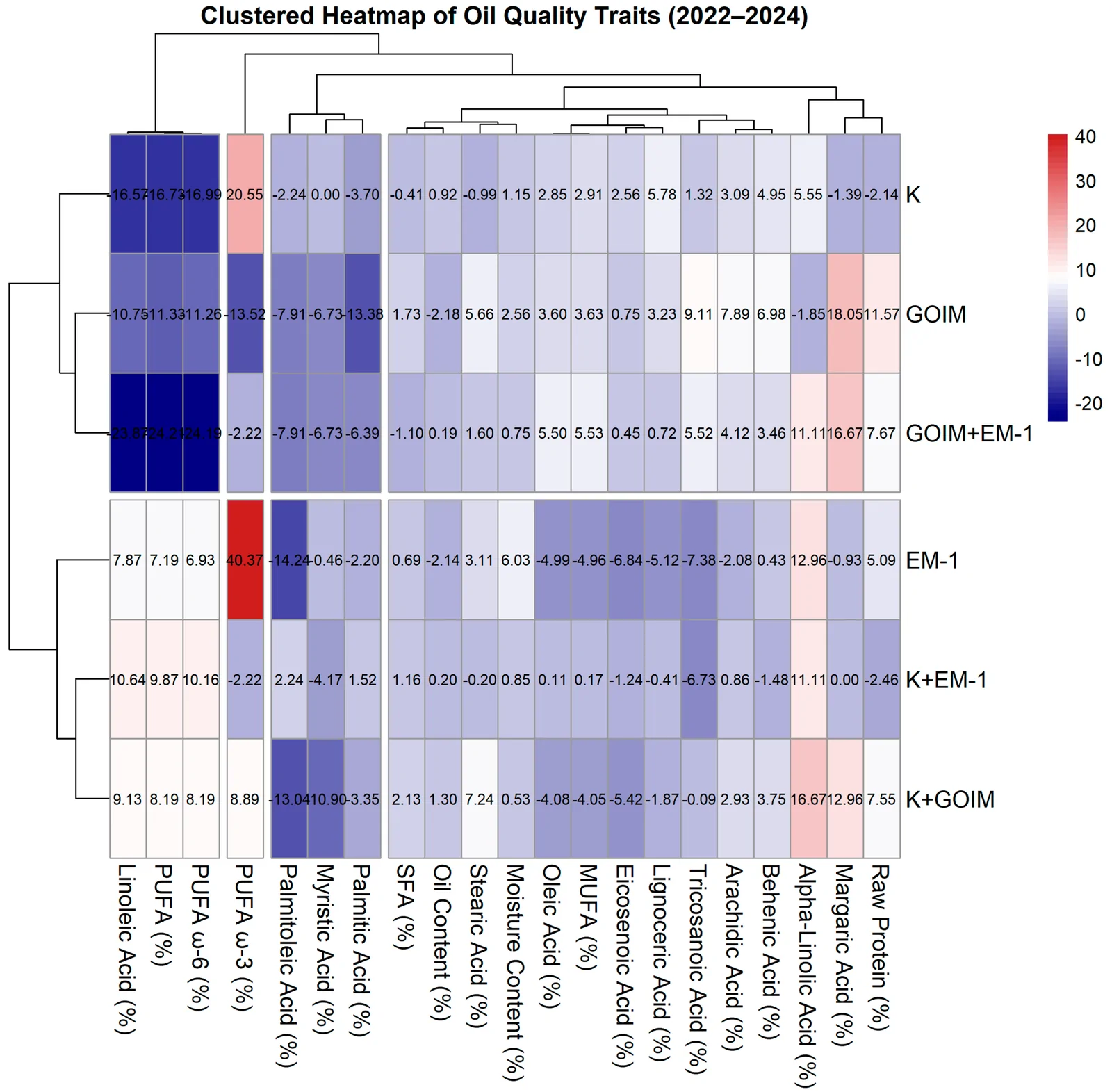
Heatmap of treatment effects on fatty acid composition in sunflower.

The percentage of palmitic acid C16:0, a type of saturated fatty acid commonly found in sunflower oil, affects the oil’s stability and shelf life. Furthermore, the levels of C16:0 were analyzed (Table 2). Sunflower oil typically contains palmitic acid at an average percentage ranging from 3.69% to 5.73%. The concentration of palmitic acid was significantly impacted by year (*p* < 0.001). Table 2 presents the ANOVA results along with the pairwise comparison post hoc tests performed using Games–Howell’s method. In 2023, this acid showed notable significance across all treatments, the mean rising from 4.40% in 2022 to 5.45%, an increase of 24%. The multivariate two-way ANOVA analyses conducted on the stearic acid (C18:0) data shown in the table indicated a significant effect of year (*p* < 0.001), as well as a notable interaction between year and fertilizer treatments (*p* = 0.009). The results of the post hoc tests, derived from pairwise comparisons using Games–Howell’s method, are presented in Table 2. The concentration of stearic acid varied annually, ranging from 2.63% to 4.10%; 2023 marked the year with the most substantial increase, followed by 2022 and 2024. Relative to 2022, the mean stearic acid in 2023 rose by 23% under K+GOIM and by 51% under K. No measurable difference was observed between the years 2022 and 2024. In the year 2022, the statistical evaluations uncovered a significant interaction between the type of fertilizer applied and year. Among the various treatments, K+GOIM achieved the numerically highest stearic acid percentages (3.29%), followed by EM-1 (3.23%), GOIM+EM-1 (3.07%) and GOIM (2.94%), although the main effect of fertilizer treatment on stearic acid was not significant (*p* = 0.14). In 2023, K (4.10%), GOIM (4.03%) and K+GOIM (4.03%) produced the most elevated concentrations of stearic acid, without significant differences among them. Lastly, in 2024, the treatments GOIM (3%) and K+EM-1 (2.84%) showed increases in stearic acid levels in the oil, with no single treatment being significantly superior. In 2023, a significant rise in myristic acid (C14:0), which was found at levels of 0.020% to 0.043% in sunflower oil, was noted across all treatments (*p* < 0.001), with the largest increases, about 43% relative to 2022, observed in the control and in the oil from K and K+EM-1. The oil from the GOIM+EM-1 treatment maintained a consistent myristic acid percentage over the years, approximately 0.03%. In 2024, the oil from K+GOIM showed the lowest myristic acid level recorded in years, around 0.02% (Table 2). Margaric acid (C17:0), a type of saturated fatty acid, showed a notable year effect (*p* < 0.001). The oils derived from the GOIM, K+EM-1, and K+GOIM treatments were the only ones to demonstrate this response. In 2023, the oils from these treatments displayed significantly elevated margaric acid levels; averaged across treatments, the content was 0.041% in 2023 compared with 0.034% in 2022 and 0.027% in 2024 (Table 2). Arachidic acid (C20:0) showed a notable year effect (*p* < 0.001) across all treatments, with the exception of the oil from K and K+EM-1 treatments, which were not statistically significant in any of the years. The level of arachidic acid in the EM-1, GOIM and K+GOIM treatments increased significantly between 2022 and 2024, by approximately 12%. No notable differences were observed in the K and K+EM-1 treatments across the years. Averaged across treatments, the arachidic acid content was lower in 2023 (0.30%) than in 2022 (0.33%) and 2024 (0.35%). However, in 2022, the arachidic acid level in the GOIM+EM-1 treatment was significantly higher at 0.37%. A significant interaction between year and fertilizer treatment was observed for this acid (*p* = 0.04). The combination of year and fertilizer treatment in 2022 yielded higher levels of arachidic acid in oil from GOIM+EM-1 (0.37%), followed by GOIM (0.33%) and K (0.33%). In 2024, the arachidic acid content ranged from 0.33% to 0.38%, with GOIM being numerically the highest, but no significant difference between treatments was detected (Table 2). In conclusion, the GOIM treatment increased both stearic and arachidic acids. K+GOIM and EM-1 also elevated stearic acid, the main saturated fatty acid of the oil. Arachidic acid was likewise increased by GOIM+EM-1 and K, whereas K slightly reduced stearic acid relative to the control (Figure 3).

#### 2.2.3. Polyunsaturated Fatty Acids

Linoleic acid (C18:2), the major polyunsaturated fatty acid of sunflower oil, was present at concentrations ranging from 3.63% to 60.52% across the three seasons (Table 3). The levels of linoleic acid were significantly affected by both year (*p* < 0.001) and fertilizer treatment (F) (*p* < 0.001), as determined through follow-up multivariate two-way ANOVA tests. Moreover, there was a notable interaction effect between year and the type of fertilizer treatment (*p* < 0.001). Averaged across treatments, the linoleic acid content was 20.3% in 2022, 59.8% in 2023 and 5.4% in 2024, which corresponds to a relative increase of 195% from 2022 to 2023 and a relative decrease of 91% from 2023 to 2024. The highest percentages of linoleic acid were recorded in 2023, when the treatment means ranged from 58.3% to 60.5%. When compared to other treatments, the oils from the EM-1 (32.1%) and K+GOIM (32.0%) treatments showed the highest linoleic acid concentrations in 2022. Conversely, the treatment GOIM+EM-1 resulted in the lowest linoleic acid content, around 10.68%. α-Linolenic acid (C18:3), a polyunsaturated fatty acid present in sunflower oil only in trace amounts, showed a significant increase in 2023 (*p* < 0.001) across all treatments, followed by 2022 and 2024. Table 3 presents the post hoc results of the multivariate ANOVA pertaining to α-linolenic acid. The interaction between year and treatment led to a notable rise in α-linolenic acid concentration in oil from K+GOIM (0.045%), then EM-1 (0.043%), GOIM+EM-1 (0.04%), and finally K+EM-1 (0.04%) (*p* < 0.001). In 2022, the lowest α-linolenic acid levels (0.03%) were recorded for the GOIM treatment and the control. In summary, both the K+GOIM (potassium combined with organic N and inorganic N) and EM-1 (effective microorganisms) treatments resulted in the highest concentrations of linoleic and alpha-linolenic acids. Averaged over the three seasons, α-linolenic acid was raised relative to the control by K+GOIM, EM-1, GOIM+EM-1, K+EM-1 and K, whereas GOIM slightly reduced it (Figure 3). Conversely, the GOIM+EM-1 (organic N and inorganic N combined with effective microorganisms) and K+EM-1 (potassium combined with effective microorganisms) enhanced alpha-linolenic acid content, even though alpha-linolenic acid comprises a smaller proportion of sunflower oil compared to linoleic acid.

### 2.3. Effect of Fertilizer Treatment and Year on Oil Stability

Saturated fatty acids (SFAs) include myristic acid, palmitic acid, margaric acid, stearic acid, arachidic acid, behenic acid, tricosanoic acid, and lignoceric acid. Monounsaturated fatty acids (MUFAs) comprise oleic acid, palmitoleic acid, and eicosenoic acid. Polyunsaturated fatty acids (PUFAs) consist of linoleic acid and alpha-linolenic acid. These categories provide important information regarding the stability of oil. Generally, higher amounts of MUFAs, especially oleic acid, tend to enhance the stability of oil. On the other hand, PUFAs and SFAs contribute to the content of essential fatty acids but may lower stability due to their greater vulnerability to oxidation. The two-way multivariate ANOVA of total MUFAs revealed a significant year effect (*p* < 0.001), a treatment effect (*p* < 0.001), and a notable interaction effect between year and fertilizer treatment (*p* < 0.001). The post hoc results are shown in Table 4 below. In 2024, MUFAs reached their highest level, averaging 85.7% across treatments, compared with 70.3% in 2022 and 29.1% in 2023. In 2022, the GOIM+EM-1 (80.0%) and K (76.7%) treatments displayed the highest MUFA levels when compared to the other treatments, as indicated by the interaction between year and fertilizer treatment. The lowest values were noted for EM-1 (58.14%) and K+GOIM (58.19%). The oils of the GOIM+EM-1 and K treatments were rich in MUFAs because they contained the highest concentrations of oleic acid, the main monounsaturated fatty acid.

The two-way multivariate ANOVA of total PUFAs revealed a significant year effect (*p* < 0.001), a treatment effect (*p* < 0.001), and a notable interaction effect between year and fertilizer treatment (*p* < 0.001). The post hoc test results, obtained via pairwise comparison utilizing Games–Howell’s method, are shown in Table 4. In terms of PUFA production, the year 2023 was by far the most productive at 59.9%, followed by 2022 at 20.4% and 2024 at 5.5%. The post hoc comparisons separated the treatments most clearly in 2022 (Games–Howell). The highest PUFA levels were recorded in the EM-1 and K+GOIM treatments in that season, at 32.14% and 32.03%, respectively. The GOIM+EM-1 treatment displayed the lowest recorded PUFA levels, approximately 10.73%. Both the K+GOIM and EM-1 treatments led to a high concentration of PUFAs in the oil, as evidenced by the elevated levels of linoleic and alpha-linolenic fatty acids. Comparable results were observed for PUFA ω-3 and PUFA ω-6 in the K+GOIM and EM-1 treatments. The subsequent multivariate two-way ANOVA analyses of SFAs indicated a significant effect of year (*p* < 0.001) and a notable interaction effect between year and fertilizer treatment (*p* = 0.008). The results of the pairwise comparison post hoc tests, obtained using Games–Howell’s method, are presented in Table 4. SFA levels were significantly elevated in 2023 compared to both 2022 and 2024. In 2022, the post hoc comparison separated the EM-1 (9.72%) and K+GOIM (9.78%) treatments, yielding the highest SFA values, although the main effect of fertilizer treatment on total SFAs was not significant (*p* = 0.18). The differences among all treatments were minimal. Treatment K exhibited the lowest amount of saturated fatty acids (SFAs), at roughly 9%. Since the treatments with EM-1 and K+GOIM yielded the highest concentrations of stearic acid, which is the main saturated fatty acid, they resulted in the greatest total SFAs compared to the other treatments. In 2022, the oils from GOIM+EM-1 and K had the highest MUFA/PUFA values, at 7.56 and 5.37, respectively; the effect of treatment on this ratio was significant (*p* = 0.01), as was its interaction with year (*p* = 0.008). A similar pattern was found for the oleic/linoleic ratio calculated from Table 1 and Table 3, which reached 7.46 under GOIM+EM-1 and 5.34 under K in the same season. In 2024, the highest values of these ratios were recorded for EM-1 at 26.0, K+GOIM at 22.3 and GOIM+EM-1 at 19.4 (Table 4).

### 2.4. Overall Impact of Fertilizer Treatments on Fatty Acid Composition Across Years

The heatmap (Figure 3) displays the impact of different fertilizer treatments on the percentage change in various fatty acids and oil components when compared to the control. For every treatment and trait, the value shown is the mean of the three annual percentage changes, each calculated relative to the unfertilized control of the same growing season. Among the treatments analyzed are effective microorganisms (EM-1), potassium (K), and organic nitrogen combined with inorganic nitrogen (GOIM), along with their combinations. Notably, the use of EM-1 on its own had the largest positive effect on ω-3 polyunsaturated fatty acids (PUFAs) and alpha-linolenic acid, yielding increases of 40.37% and 12.96%, respectively, compared to the control (indicated by red areas), and a small increase in linoleic acid of 7.87%. Additionally, the change in K+GOIM compared to the control increased alpha-linolenic acid by 16.67%, PUFA (ω-3) by 8.89%, and linoleic acid by 9.13%. In contrast, the GOIM+EM-1 treatment experienced the greatest reductions in linoleic acid, PUFAs, and PUFA (ω-6) with decreases of 23.87%, 24.21%, and 24.19%, respectively (signified by blue areas).

Moderate variations were observed in oleic acid, an essential monounsaturated fatty acid, across the different treatments. Compared with the control, oleic acid increased most under GOIM+EM-1, by 5.5%, followed by GOIM at 3.6% and K at 2.9%, whereas K+EM-1 was practically unchanged at 0.1% and EM-1 and K+GOIM reduced oleic acid by 5.0% and 4.1%. α-Linolenic acid, present only in trace amounts, increased under every treatment except GOIM, most strongly under K+GOIM at 16.7%, EM-1 at 13.0% and GOIM+EM-1 and K+EM-1 at 11.1%. Overall, EM-1 was the most effective treatment for raising ω-3 fatty acids, whereas GOIM applied alone or combined with EM-1 lowered ω-6 fatty acids by 11.3% and 24.2% and produced the two largest increases in oleic acid, 3.6% and 5.5%.

### 2.5. AMMI PCA of Treatment Effects on Fatty Acid Composition over Three Years

**Figure 4:**
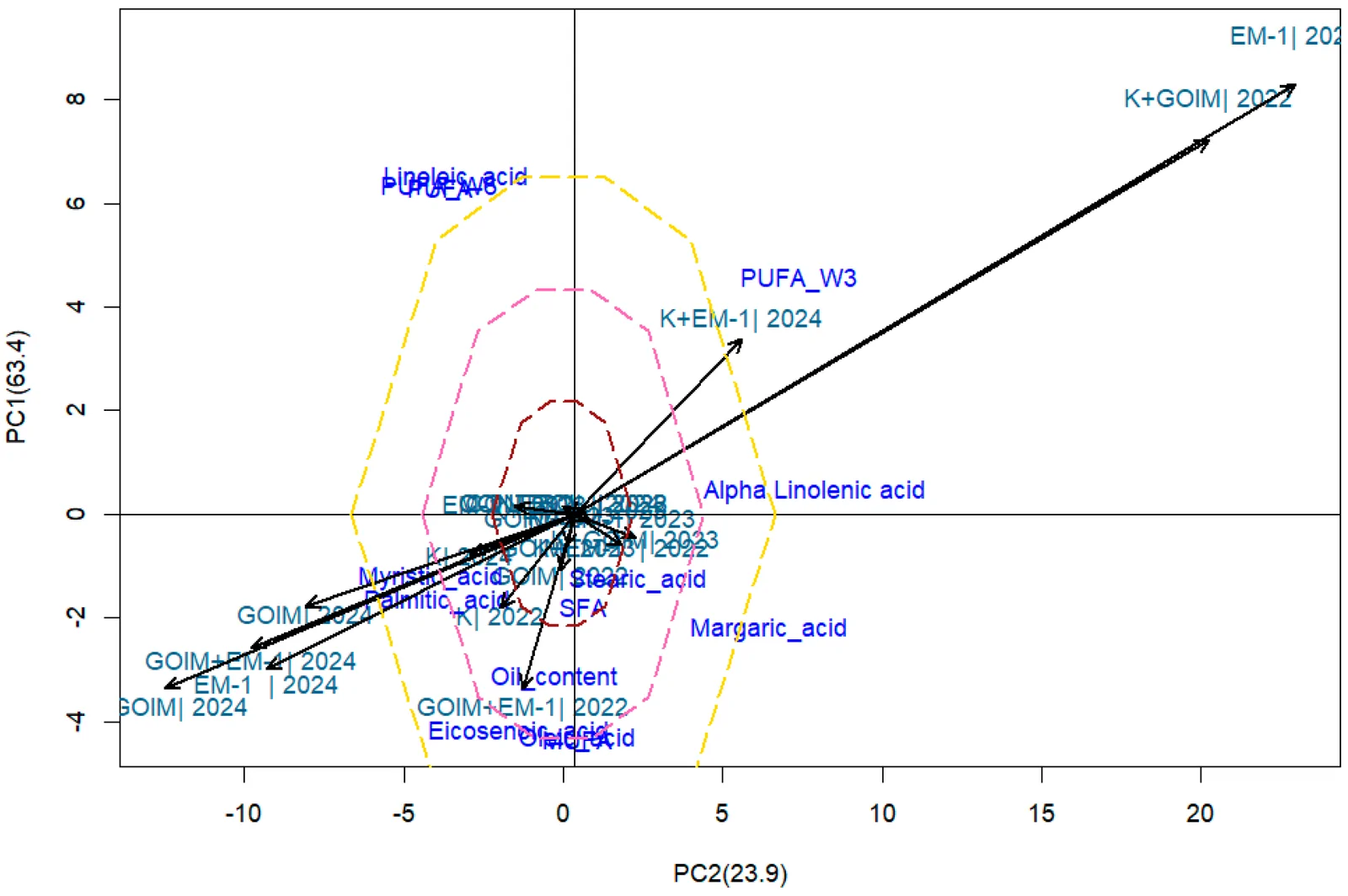
Biplot analysis of oil quality parameters in response to different fertilizer treatments and years using the AMMI model. Black arrows run from the origin to each fertilizer treatment × year combination, labelled in grey-blue as “treatment|year”; a longer arrow indicates a stronger contribution to the interaction, and traits, labelled in blue without arrows, are positively associated with the combinations lying in the same direction. The dashed contours, dark red, pink and gold from the innermost outwards, group combinations with similar effects on the oil quality parameters.

The analysis results indicate that fatty acid composition and oil quality are significantly influenced by both the year and fertilizer applications. The year factor is statistically significant (*p* = 0.001), indicating that the measured parameters of oil quality are significantly influenced by the environmental conditions of various years. Furthermore, the replicates within each year also demonstrate highly significant differences (*p* < 0.001), suggesting that the variation among repetitions in the same year is not a result of arbitrary chance. This underscores the necessity of accounting for year-to-year variability in oil composition analysis. Furthermore, the fertilizer treatments are highly significant (*p* < 0.001), which confirms that the quality of the oil is substantially influenced by the type of fertilizer used. This finding suggests that specific fertilizers are more effective in improving oil composition than others, underscoring the significance of selecting the appropriate fertilizer type to enhance oil yield and composition. The interaction between fertilizer treatments and year is also highly significant (*p* < 0.001), suggesting that the impact of fertilizers on oil quality is dependent upon the unique conditions of each year. This interaction implies that the efficacy of fertilizers may fluctuate as a result of external factors, including soil conditions, weather, or other environmental influences. Consequently, optimizing oil quality outcomes necessitates appropriate fertilizer application strategies to the unique conditions of each year. The AMMI model’s biplot analysis of the oil quality parameters in response to various fertilizer treatments and years provided valuable insights into the interactions between treatment and year (Figure 4). Principal Component 1 (PC1) accounted for 63.4% of the total variance, while Principal Component 2 (PC2) accounted for 23.9%. Combined, these components accounted for 87.3% of the total variability in the dataset. This implies that these two principal components captured the majority of the variability in the oil’s fatty acids. The contour lines help visualize the clustering of different fertilizer × year combinations. For instance, treatments within the same contour lines exhibit similar effects on oil quality parameters, while those falling outside of these lines display more distinct influences. This suggests that the specific year and type of fertilizer can lead to similar or divergent effects on oil composition depending on the treatment. The results (Figure 4) indicate that the polyunsaturated fatty acid (PUFA ω-3) and alpha-linolenic acid contents of the critical oil quality traits were significantly and positively influenced by the combination of the K+GOIM fertilizer in 2022 and the EM-1 fertilizer in 2022. This treatment is positively correlated with an increase in these beneficial oil components, which are crucial for the nutritional quality of oil, as indicated by the direction and magnitude of the arrows for these parameters. In comparison to other fertilizer × year combinations, the significant impact of these fertilizer treatments on enhancing oil composition is evident in their position far from the center of the biplot in 2022. However, treatments such as GOIM in 2024 and EM-1 in 2024 were positioned closer to the center of the biplot, indicating that these combinations had a more moderate or balanced effect on oil quality parameters. These interventions may not result in substantial changes in the measured oil characteristics, but rather in the preservation of a consistent oil quality profile. The results suggest that the most promising treatments for improving oil quality in 2022 are the combination of K+GOIM and EM-1 fertilizers, particularly in terms of increasing the levels of polyunsaturated fatty acids such as PUFA ω-3. This implies that the nutritional quality of oils can be substantially enhanced by the optimal application of fertilizers and favorable environmental conditions during specific years. These results emphasize the significance of selecting the appropriate fertilizer treatments in conjunction with environmental factors to optimize oil composition.

## 3. Discussion

The current study aimed to evaluate the influence of different fertilizer treatments on sunflower seed quality and oil productivity across multiple growing seasons. The findings, supported by multivariate ANOVA, heatmap clustering, and AMMI PCA, revealed significant differences among treatments, indicating a strong interaction between fertilizer application and year environment.

### 3.1. Impact of Fertilizer Applications on the Nutritional Composition (Oil and Protein) of Sunflower Seeds

The highest oil contents in the drought season of 2022 were recorded under the K and K+EM-1 treatments, although they exceeded the control by less than one percentage point. Potassium’s central role in carbohydrate translocation and lipid biosynthesis in developing seeds offers a plausible explanation for this ranking. Potassium enhances photosynthetic efficiency and promotes the transport of assimilates from source leaves to the capitulum, providing substrates required for fatty acid synthesis [31,32]. Under rainfed conditions, this role becomes particularly important because potassium also regulates stomatal conductance and osmotic adjustment, thereby sustaining metabolic activity during water limitation and helping to preserve oil quality [33]. Consequently, the higher oil accumulation observed in the present study reflects both improved carbon supply and enhanced stress tolerance induced by potassium nutrition. The interaction between potassium and EM-1 in sunflower oil quality has not been extensively studied. In the present experiment, K+EM-1 did not exceed K alone in any of the three seasons, so these data provide no evidence that adding EM-1 to potassium improved oil accumulation further. EM-1 likely enhanced potassium availability in the rhizosphere through microbial solubilization and improved root development, resulting in greater nutrient uptake efficiency. Similar combined effects of microorganisms and chemical fertilizers have been reported in other oilseed crops; for instance, *Azotobacter* and mycorrhiza combined with mineral fertilization increased oil content and yield in safflower and soybean [34,35]. Moreover, EM-1 improves nutrient uptake, plant growth, and nutritional quality in crops such as tomato and mung bean [36,37] and enhances plant resilience under stressful conditions [38]. These findings support the hypothesis that effective microorganisms contributed to the improved oil accumulation and stability observed under the K+EM-1 treatment. Regarding protein content, the higher values recorded under the GOIM, K+GOIM and GOIM+EM-1 treatments, all of which contained a combined organic and inorganic nitrogen source, may be related to nutrient availability and microbial activity. Because the pelleted manure also supplied phosphorus and potassium (Section 4.3), these responses cannot be attributed to nitrogen alone; they reflect the combined supply of the three nutrients together with the organic matter itself. Nitrogen is a key structural component of amino acids and proteins, and its supply is more efficiently regulated when organic sources are combined with inorganic forms due to gradual mineralization [39]. EM-1 further enhances nitrogen availability through biological processes such as nitrogen fixation, nutrient solubilization, and stimulation of root growth, thereby supporting metabolic pathways related to protein synthesis. Potassium also contributes by activating enzymes involved in protein formation and improving internal nutrient transport, complementing nitrogen’s role. This interpretation is consistent with previous studies showing that manure combined with chemical fertilizers increases seed yield, protein content, and oil production in sunflower [40], and that potassium and nitrogen application improves physiological performance and protein accumulation under water stress [12,41]. The inverse relationship observed between protein and oil content across treatments reflects a physiological trade-off in seed resource allocation. When nitrogen availability is high, carbon skeletons are preferentially diverted toward amino acid and protein biosynthesis at the expense of lipid accumulation. Such antagonism between protein and oil content has been widely reported in oilseed crops, where increases in protein concentration are often associated with reductions in oil content [42,43]. This trade-off explains the reduced oil content observed under nitrogen-enriched treatments such as K+GOIM and GOIM+EM-1, despite their positive effects on protein concentration. Overall, the present results indicate that potassium-based fertilization, particularly when combined with EM-1, is more effective for enhancing oil content and maintaining oil quality under rainfed conditions, whereas integrated nitrogen and organic matter strategies mainly promote protein accumulation. These findings highlight the importance of selecting fertilizer combinations according to production objectives, whether targeting oil yield or protein enrichment under drought-prone environments.

### 3.2. Effect of Fertilizer Treatment on the Fatty Acid Composition of Sunflower Oil

The three growing seasons produced markedly different fatty acid profiles. Averaged across treatments, oleic acid reached 69.9% in 2022, 28.9% in 2023 and 85.3% in 2024, whereas linoleic acid followed the opposite order: 20.3%, 59.8% and 5.4%. Palmitic and stearic acids were highest in 2023, at 5.45% and 3.89%, lowest in 2024, at 3.82% and 2.80%, and intermediate in 2022, at 4.40% and 2.98%. The drought season in 2022 therefore did not shift the profile towards linoleic acid. Compared with the wetter 2023 season, it produced 2.4 times more oleic acid and about one third of the linoleic acid, together with lower palmitic and stearic acid contents. In contrast, compared with 2024, it produced less oleic acid and more linoleic, palmitic and stearic acid, so 2022 was intermediate between the two other seasons for every fatty acid examined. The mean temperature in July and August, the period covering flowering and seed filling, ranked the three seasons in the same order as their oleic acid content: 22.5 °C in 2023, 23.8 °C in 2022 and 24.7 °C in 2024 (Table 5). This is consistent with the temperature dependence of oleate desaturase, the activity of which declines as temperature rises during seed filling, so that a smaller proportion of oleic acid is converted into linoleic acid. Drought-induced shifts in the oleic–linoleic balance have also been reported, generally as increases in palmitic and linoleic acids together with a decrease in oleic acid [44,45], but in the present experiment, this pattern appeared only in the comparison between 2022 and 2024 and was reversed in the comparison with the cooler 2023 season, which indicates that the thermal regime of the seed-filling period rather than the water supply alone governed the ranking of the seasons. The oleic acid content measured in 2023 deserves particular attention because ES Emeric is a high-oleic hybrid for which the breeder reports an oleic acid content of 90–91%. The treatment means in that season ranged from 28.2% to 30.4%, a profile characteristic of a standard linoleic hybrid, whereas the values in 2024, from 81.3% to 87.2%, correspond to the expected genotypic profile. All seven treatments and all three replicates in 2023 behaved the same way, so the departure is a property of season, not of fertilization. The cool seed-filling period in 2023 is the most plausible contributing factor, but a difference of about two degrees is not usually reported to produce a shift of this magnitude in genotypes carrying the high-oleic FAD2-1 allele. This observation should therefore be regarded as a limitation of the present dataset, and confirmation on an independent seed lot would be required before a physiological mechanism is proposed. Contrasting responses to water deficit have also been observed depending on the developmental stage at which the stress occurs. For instance, Flagella et al. [46] reported increased oleic acid when drought occurred between flowering and maturity, highlighting that stress timing is a critical determinant of fatty acid composition. The partial agreement with Ali et al. [47], who observed increased stearic acid and reduced linolenic acid under drought, further suggests that drought and heat stress affect multiple enzymatic steps of fatty acid biosynthesis rather than a single component. Fertilization strategies exerted a strong regulatory effect on fatty acid profiles. The GOIM+EM-1 treatment produced the highest oleic acid concentration, indicating stimulation of monounsaturated fatty acid (MUFA) synthesis. This response may be attributed to improved nitrogen availability and enhanced microbial activity, which support enzymatic reactions involved in lipid metabolism. The combined supply of organic and inorganic nitrogen likely ensured both sustained and readily available nutrients during seed filling, while EM-1 improved nutrient uptake and root development. This interpretation differs from Alzamel et al. [48], who observed reduced oleic acid under compost plus biofertilizer, emphasizing that biological inputs interact strongly with mineral nutrition and environmental conditions. Potassium application significantly increased oleic and eicosenoic acids, supporting its role in enzyme activation, photosynthesis, and assimilate transport to developing seeds. Potassium fertilization has been shown to improve sunflower oil quality and maintain fatty acid stability under drought stress [31,32,33]. In the present study, potassium-based treatments also contributed to buffering the negative impact of climatic stress on oil composition. The highest linoleic and α-linolenic acid contents, together with moderate oleic acid levels, were observed under the K+GOIM and EM-1 treatments, leading to increased polyunsaturated fatty acids (PUFAs). This response may be linked to enhanced activity of desaturase enzymes stimulated by organic inputs and improved soil biological activity [49]. Organic manure has been shown to increase unsaturated fatty acids in oilseed flax [49], supporting the view that organic matter promotes PUFA synthesis through improved nutrient supply and microbial stimulation. The negative relationship between linolenic acid and both oleic and linoleic acids reflects its synthesis from linoleic acid via successive desaturation steps [49]. Distinct treatment responses observed in 2022 further illustrate the regulatory role of biological inputs. GOIM+EM-1 increased oleic acid while reducing linoleic acid, whereas EM-1 enhanced linoleic and α-linolenic acids but lowered oleic acid. Effective microorganisms likely influenced fatty acid synthesis by stimulating enzyme activity and nutrient absorption, consistent with observations in sesame oil [50]. The GOIM treatment moderately increased both saturated and unsaturated fatty acids, confirming that integrated nitrogen management supports lipid biosynthesis but requires combination with potassium or EM-1 to optimize specific fatty acid fractions. A response in the same direction was reported by Akbari et al. [30], whose combination of farmyard manure with mineral nitrogen and bacterial inoculation raised unsaturated and lowered saturated fatty acid fractions, although the absolute values are not comparable with those obtained here because that experiment used a standard linoleic cultivar, covered a single irrigated season and did not include potassium as a factor. Among all treatments, GOIM+EM-1 produced the highest oleic acid content, whereas K+GOIM maximized linoleic acid, identifying them as the most effective strategies for enhancing MUFAs and PUFAs, respectively. These findings are consistent with Shoghi-Kalkhoran et al. [51], who reported the highest linoleic acid levels under combined farmyard and chemical fertilization and the highest oleic acid under mixed mineral and organic fertilization. The superior MUFA/PUFA and oleic/linoleic ratios recorded under GOIM+EM-1, followed by potassium and K+EM-1, indicate improved oxidative stability of sunflower oil [52]. Therefore, integrating nitrogen management with microbial or potassium inputs allows targeted modulation of sunflower oil quality under drought-prone conditions.

### 3.3. Integrated Interpretation of Oil-Related Responses to Fertilizer Treatment

The combined clustering heatmap analysis and AMMI PCA demonstrated that sunflower oil composition was jointly regulated by fertilizer strategy and inter-annual environmental variability. Rather than showing uniform treatment effects, these multivariate approaches revealed distinct treatment–year groupings, indicating that the response of fatty acid composition to fertilization depended strongly on climatic conditions during each growing season. This supports previous findings that fertilizer management is a major determinant of sunflower oil quality and also highlights that its effectiveness is modulated by environmental factors [53,54,55,56]. Heatmap clustering showed that the K+GOIM and EM-1 treatments were most closely associated with increased polyunsaturated fatty acids, particularly linoleic and α-linolenic acids, suggesting that these strategies preferentially promote desaturation pathways under favorable or moderately stressful conditions. In contrast, the GOIM+EM-1 combination clustered with higher oleic acid and MUFA levels, indicating a shift toward monounsaturated fatty acid synthesis. This divergence implies that nutrient supply and microbial activity influenced the balance between competing fatty acid biosynthetic pathways rather than uniformly increasing all unsaturated fractions. AMMI PCA further clarified the role of environmental variability by revealing strong treatment × year interactions. In 2022, when higher temperatures and reduced precipitation intensified drought stress, the K+GOIM and EM-1 treatments were positioned closer to PUFA-related traits, particularly α-linolenic acid, indicating that these strategies were more effective in promoting PUFA accumulation under stress conditions. However, in 2023 and 2024, when climatic conditions were less severe, this association weakened, demonstrating that the impact of these fertilizer treatments on fatty acid composition was not stable across years. This instability suggests that their effectiveness is sensitive to temperature and water availability during seed filling, when fatty acid desaturation is most active. These results highlight that fertilizer strategies differ not only in their capacity to modify oil quality but also in their stability across contrasting climatic conditions. Treatments favoring PUFA accumulation (K+GOIM and EM-1) appeared more responsive to drought-related environmental cues, whereas GOIM+EM-1 promoted oleic acid and MUFAs more consistently. Therefore, adaptive fertilizer management that considers inter-annual climatic variability is essential for optimizing sunflower oil quality, particularly under rainfed conditions where temperature and precipitation strongly regulate fatty acid metabolism.

## 4. Materials and Methods

The current research was carried out in the experimental plot at the Hungarian University of Agriculture and Life Sciences (MATE) located in Gödöllő, Hungary, over the three growing seasons of 2022, 2023 and 2024. The site in Gödöllő is positioned at a latitude of 47°35′41″ N and a longitude of 19°22′07″ E. The soil within the experimental area is classified as sand-based brown forest soil (Chromic Luvisol). It falls under the sandy loam textural category. Agronomically, the soil is neutral sandy, with varying clay content. The soil structure faces issues related to compaction. Due to the high content of sand, the water retention characteristics are inadequate. The soil has been subject to the effects of drought.

### 4.1. Meteorological Data of the Experimental Period

The meteorological data, essential for understanding environmental factors, were carefully collected using a calibrated meteorological tower positioned within the experimental site to ensure optimal data collection at 10 min intervals.

Climatic data from May to September over the years 2022 to 2024 revealed significant inter-annual variability in temperature, humidity, and rainfall, all of which can critically influence crop growth and productivity (Table 5). In 2022, the region experienced a hot and dry summer, with average temperatures peaking in July at 23.7 °C and in August at 23.8 °C. Total rainfall from May to September was 209.7 mm, but only 125.1 mm of this fell between May and August, the period covering vegetative growth, flowering and seed filling. The remaining 84.6 mm fell in September, after physiological maturity, and therefore did not contribute to crop water supply. Humidity levels also dropped significantly in July (48.58%), potentially exacerbating water stress. In contrast, 2023 recorded cooler temperatures in the early months, with a peak of 22.82 °C in July, and considerably higher rainfall, particularly in May (78.3 mm) and August (113.7 mm). Humidity remained relatively high, contributing to a more humid and wetter growing environment that could favor plant development but also increase the risk of fungal infections. The year 2024 showed a balance between both extremes, with high temperatures in July (24.72 °C) and August (24.59 °C) and moderate rainfall across the season, most notably in June (65.1 mm) and September (82.8 mm). Humidity remained within moderate ranges, suggesting a potentially favorable climate for sunflower growth, especially in terms of oil accumulation and drought resilience. Rainfall between May and August was 269.6 mm in 2023 and 159.2 mm in 2024. The critical growth period of 2022 therefore received 54% less rainfall than that of 2023 and 21% less than that of 2024, which makes 2022 the driest of the three seasons.

### 4.2. The Studied Crop

The experimental trials were carried out on the sunflower hybrid “ES Emeric”, a mid-early high-oleic hybrid bred by Lidea Seeds (Lescar, France) and registered in Italy in 2021. According to the breeder’s description, the hybrid matures in 106–110 days, reaches a seed oil content of 49–51% and an oleic acid content of 90–91% of total fatty acids, is tolerant to imazamox under the Clearfield Plus production system, shows good drought tolerance and is highly resistant to rust and Verticillium. The recommended sowing rate is 45,000–55,000 seeds per hectare. The seeds, treated with Fludioxonil + Metalaxyl, had a thousand-kernel weight of approximately 101.4 g. The soil preparation involved moldboard plowing to a depth of 30 cm, followed by harrowing and shallow tine cultivation to create an optimal seedbed. Sunflowers were planted using a MaterMacc MS4100 planting machine (MaterMacc, San Vito al Tagliamento, Italy) at a depth of 3 cm, with a spacing of 24 cm between plants and 75 cm between rows, corresponding to a plant density of approximately 55,000 plants ha^−1^. The field, covering 1 hectare, was divided into four parcels of 2500 m^2^ each, following a crop rotation of winter wheat, sunflower, maize and green manure. Sowing was coordinated after rainfall when the soil temperature exceeded 12 °C. All trials relied only on rainfed conditions. The sunflower crop was sown on 5 May 2022, 25 April 2023, and 2 May 2024.

### 4.3. Details of Treatments

The experiment was arranged as a randomized complete block design with three blocks and was carried out over three consecutive growing seasons, 2022, 2023 and 2024, under rainfed conditions in Hungary (Table 6). Seven treatments were compared, namely, an unfertilized control, mineral potassium (K), combined organic and inorganic nitrogen (GOIM), effective microorganisms (EM-1), and the three two-way combinations K+GOIM, K+EM-1 and GOIM+EM-1. All fertilizer rates are expressed on an active ingredient basis. Potassium was applied at 100 kg K_2_O ha^−1^ as granular potassium sulphate containing 50% K_2_O, which corresponds to 200 kg of product per hectare. The GOIM treatment supplied 100 kg N ha^−1^, half of it from a mineral and half from an organic source. The mineral half was 50 kg N ha^−1^ applied as 147 kg ha^−1^ of ammonium nitrate containing 34% N, and the organic half was 50 kg N ha^−1^ applied as 1250 kg ha^−1^ of a commercial pelleted cattle manure with a declared content of 4% N, 4% P_2_O_5_ and 4% K_2_O. Effective microorganisms were applied at 40 L ha^−1^, the rate specified by the manufacturer, as the commercial preparation EM-1, which contains photosynthetic bacteria, lactic acid bacteria, yeasts, actinomycetes and fermenting fungi. All rates were converted proportionally to the area of the individual experimental plots. No separate phosphorus fertilizer was applied to any treatment. The pelleted manure, however, is not a nitrogen-only source: applied at 1250 kg ha^−1^, it also supplied 50 kg P_2_O_5_ ha^−1^ and 50 kg K_2_O ha^−1^, so the three treatments containing GOIM received phosphorus and additional potassium alongside the intended nitrogen. Expressed as kg ha^−1^ of N, P_2_O_5_ and K_2_O, the resulting nutrient balance was 0-0-0 for the control and for EM-1, 0-0-100 for K and for K+EM-1, 100-50-50 for GOIM and for GOIM+EM-1, and 100-50-150 for K+GOIM. GOIM is therefore a combined NPK treatment rather than a nitrogen treatment, and the potassium supply of K+GOIM exceeded that of K by 50%. This overlap between the fertilization factors is taken into account in the interpretation of the results.

The 1 ha experimental field was divided into four parcels of 2500 m^2^ serving the crop rotation. In each season, the parcel carrying sunflower was subdivided into 21 experimental plots, corresponding to seven treatments in three randomized blocks, each plot having a net area of 1.8 m^2^ from which the plants used for measurement were taken. Treatments were randomly assigned within each block to minimize the effects of spatial variability. The seven treatments represent an incomplete factorial arrangement of three fertilization factors, namely, potassium, combined organic and inorganic nitrogen, and effective microorganisms. The arrangement comprised the untreated control, each factor applied alone, and all three two-way combinations, whereas the three-way combination K+GOIM+EM-1 was not included. This structure allowed both the individual and the combined effects of the chemical and biological inputs to be assessed, and conducting the study over three seasons allowed treatment responses to be examined under the contrasting climatic conditions characteristic of rainfed agriculture in the region.

### 4.4. Details of the Collection of Experimental Data

The crop was harvested annually from late September to early October. The process started with collecting the sunflower heads, followed by cutting the stalks with sickles. The heads were then sun-dried for approximately one week and threshed to separate the seeds from the heads. The seeds were dried, cleaned and weighed. Seed quality was assessed on cleaned seed samples taken from each plot. All seed quality determinations were carried out at the MATE Központi Vizsgálólaboratórium in Kaposvár, a testing laboratory accredited under registration number NAH-1-1935/2024. Seed moisture content was determined according to MSZ EN ISO 665:2020 [57] and MSZ ISO 6496:2001 [58] using a Memmert UFE 500 drying oven (Memmert GmbH + Co.KG, Schwabach, Germany), crude protein content according to MSZ EN ISO 5983-2:2009 [59] using a Foss Tecator digestion unit and a Foss Kjeltec 8400 analyzer (Foss, Hilleroed, Denmark), and oil content according to MSZ ISO 7009:1983 [60] by Soxhlet extraction on a FALC BE6 unit (Falc Intruments s.r.l., Treviglio, Italy). The fatty acid profile of the seeds was determined according to MSZ EN ISO 12966-2:2011 [61] by gas chromatography on a Shimadzu 2010 instrument (Shimadzu, Kyoto, Japan), and the individual fatty acids from C14:0 to C24:0 are reported as relative percentages of total fatty acid methyl esters. Agronomic yield components were also recorded in the same trial but are reported elsewhere because the present study is concerned with seed quality and oil composition.

### 4.5. Statistical Analysis

Data were analyzed with IBM SPSS V27 and RStudio (v. 2026.04.0) [62], using the R packages RcmdrMisc, Nortest, Car and Ggplot2 for analysis and visualization. Normality was tested with the Shapiro–Wilk test and homogeneity of variances with the Levene test. The effects of fertilizer treatment and growing season on seed quality and on the fatty acid composition of the oil were first examined by two-way multivariate analysis of variance, with Wilks’ lambda as the omnibus test across the correlated response variables. Each response variable was then analyzed by univariate two-way analysis of variance with fertilizer treatment, year and their interaction as fixed effects. Where the assumption of homogeneity of variances was not met, the robust Welch analysis of variance was applied instead. Pairwise comparisons were made with the Games–Howell post hoc test. Heatmap clustering and AMMI principal component analysis were used to illustrate how treatment responses varied across the three growing seasons. The AMMI analysis was carried out with the AMMI function of the R package agricolae [63], which fits the additive main effects of fertilizer treatment and year and then decomposes the residual interaction matrix by principal component analysis, the resulting interaction principal component axes being displayed in the biplot.

## 5. Conclusions

This study assessed the effects of integrated inorganic, organic, and biological fertilization strategies on sunflower oil composition over three consecutive growing seasons under rainfed conditions in Hungary. The results indicate that combined fertilizer applications exert a stronger influence on fatty acid profiles than single-input strategies and that their effects vary according to inter-annual climatic conditions. In particular, the integration of potassium with combined organic and inorganic nitrogen (K+GOIM), as well as the application of effective microorganisms (EM-1), was associated with increased proportions of polyunsaturated fatty acids, notably linoleic and α-linolenic acids, under drought-affected conditions. Sunflower oil enriched in linoleic and α-linolenic acids is of nutritional relevance because these essential fatty acids contribute to cardiovascular health and are precursors of biologically active lipid mediators. Their accumulation therefore represents not only an improvement in oil quality but also an enhancement in its dietary value. The present findings are derived from a single field experiment conducted at one site and under the specific soil and climatic conditions of Hungary. In addition, the organic component of the GOIM treatments supplied phosphorus and potassium as well as nitrogen, so the responses recorded for those treatments reflect a combined nutrient supply rather than nitrogen alone. Consequently, the observed treatment effects reflect localized responses and may not be directly transferable to other agroecological regions. Validation of these results through multi-site and multi-year investigations is required to determine the consistency of integrated fertilizer strategies and to clarify the underlying physiological and microbial mechanisms regulating fatty acid composition in sunflower. Three features distinguish these findings from the earlier literature on integrated nutrient management in sunflower. They were obtained on a high-oleic hybrid rather than on a standard linoleic genotype, they include potassium and its combination with effective microorganisms as experimental factors, and they cover three consecutive seasons, which made it possible to show that the effects of fertilization on the fatty acid profile were not stable across contrasting thermal and rainfall regimes. Overall, this work provides evidence that integrated fertilizer management can serve as a practical approach for modulating sunflower oil quality in environments characterized by variable rainfall and periodic drought, thereby supporting the development of adaptive nutrient management strategies for oilseed production.

## Figures and Tables

**Table 1:** Post hoc results of fertilizer effects on monounsaturated fatty acid composition over years.

		C20:1	C18:1
Treatment	Year	Eicosenoic Acid	Oleic Acid
CONTROL	2022	0.26 ± 0.00 ^A*bc^	70.92 ± 0.122 ^B*bc^
CONTROL	2023	0.14 ± 0.01 ^A*a^	28.5 ± 3.649 ^C*a^
CONTROL	2024	0.273 ± 0.012 ^A*a^	84.25 ± 2.659 ^A*a^
EM-1	2022	0.207 ± 0.003 ^B*d^	57.84 ± 2.667 ^B*cd^
EM-1	2023	0.14 ± 0.00 ^C*a^	28.493 ± 4.294 ^C*a^
EM-1	2024	0.273 ± 0.006 ^A*a^	87.187 ± 1.315 ^A*a^
GOIM	2022	0.26 ± 0.00 ^bc^	73.05 ± 0.182 ^B*a^
GOIM	2023	0.15 ± 0.00 ^a^	30.397 ± 1.438 ^C*a^
GOIM	2024	0.26 ± 0.00 ^a^	85.2 ± 1.389 ^A*a^
GOIM+EM-1	2022	0.267 ± 0.006 ^A*b^	79.653 ± 1.758 ^B*ab^
GOIM+EM-1	2023	0.14 ± 0.00 ^B*a^	29.02 ± 3.222 ^C*a^
GOIM+EM-1	2024	0.27 ± 0.01 ^A*a^	86.243 ± 1.563 ^A*a^
K	2022	0.28 ± 0.00 ^A*a^	76.25 ± 0.885 ^B*a^
K	2023	0.14 ± 0.00 ^B*a^	28.21 ± 3.575 ^C*a^
K	2024	0.273 ± 0.012 ^A*a^	85.987 ± 1.392 ^A*a^
K+EM-1	2022	0.255 ± 0.005 ^A*c^	73.95 ± 1.09 ^A*ab^
K+EM-1	2023	0.14 ± 0.00 ^A*a^	28.36 ± 4.233 ^B*a^
K+EM-1	2024	0.247 ± 0.032 ^A*a^	81.327 ± 9.749 ^A*a^
K+GOIM	2022	0.208 ± 0.003 ^B*d^	57.89 ± 1.137 ^B*d^
K+GOIM	2023	0.143 ± 0.006 ^C*a^	29.4 ± 1.532 ^C*a^
K+GOIM	2024	0.277 ± 0.006 ^A*a^	86.76 ± 1.075 ^A*a^
*p* values	T	0.16	<0.001
*p* values	Y	<0.001	<0.001
*p* values	T × Y	0.04	<0.001

Means are presented ± SD (*n* = 3). Different letters denote statistically significant year effects (capital letters) and treatment effects (small letters). They begin in order, with letter (a/A) being the most significant. The star symbol (*) denotes the statistical interaction between two factors (year × fertilizer treatment). T: treatment; Y: year.

**Table 2:** Post hoc results of fertilizer effects on saturated fatty acid composition over years.

		C14:0	C16:0	C17:0	C18:0	C20:0
Treatment	Year	Myristic Acid	Palmitic Acid	Margaric Acid	Stearic Acid	Arachidic Acid
CONTROL	2022	0.03 ± 0.00 ^B*a^	4.703 ± 0.257 ^A*a^	0.03 ± 0.00 ^A*a^	2.81 ± 0.165 ^B*ab^	0.315 ± 0.005 ^AB*b^
CONTROL	2023	0.043 ± 0.006 ^A*a^	5.463 ± 0.492 ^A*a^	0.04 ± 0.01 ^A*a^	3.82 ± 0.562 ^A*a^	0.287 ± 0.032 ^B*a^
CONTROL	2024	0.0267 ± 0.006 ^B*a^	3.87 ± 0.147 ^B*a^	0.027 ± 0.006 ^A*a^	2.82 ± 0.175 ^B*a^	0.347 ± 0.015 ^A*a^
EM-1	2022	0.033 ± 0.003 ^AB*a^	4.588 ± 0.296 ^A*a^	0.032 ± 0.006 ^A*a^	3.227 ± 0.236 ^AB*ab^	0.31 ± 0.022 ^AB*b^
EM-1	2023	0.043 ± 0.006 ^A*a^	5.477 ± 0.573 ^AB*a^	0.037 ± 0.006 ^A*a^	3.70 ± 0.477 ^A*a^	0.273 ± 0.029 ^B*a^
EM-1	2024	0.023 ± 0.006 ^B*a^	3.7 ± 0.07 ^B*a^	0.027 ± 0.006 ^A*a^	2.753 ± 0.031 ^B*a^	0.347 ± 0.015 ^A*a^
GOIM	2022	0.03 ± 0.00 ^B*a^	4.25 ± 0.036 ^B*a^	0.04 ± 0.00 ^A*a^	2.94 ± 0.026 ^B*a^	0.337 ± 0.006 ^AB*ab^
GOIM	2023	0.04 ± 0.00 ^A*a^	5.28 ± 0.147 ^A*a^	0.043 ± 0.006 ^A*a^	4.03 ± 0.171 ^A*a^	0.31 ± 0.017 ^B*a^
GOIM	2024	0.023 ± 0.006 ^B*a^	3.82 ± 0.036 ^C*a^	0.03 ± 0.00 ^B*a^	3.013 ± 0.188 ^B*a^	0.377 ± 0.031 ^A*a^
GOIM+EM-1	2022	0.03 ± 0.00 ^A*a^	3.983 ± 0.119 ^B*a^	0.04 ± 0.00 ^A*a^	3.0733 ± 0.085 ^AB*ac^	0.367 ± 0.025 ^A*a^
GOIM+EM-1	2023	0.04 ± 0.01 ^A*a^	5.417 ± 0.477 ^A*a^	0.037 ± 0.006 ^AB*a^	3.775 ± 0.605 ^A*a^	0.283 ± 0.032 ^B*a^
GOIM+EM-1	2024	0.023 ± 0.006 ^A*a^	3.753 ± 0.064 ^B*a^	0.027 ± 0.006 ^B*a^	2.723 ± 0.178 ^B*a^	0.337 ± 0.023 ^AB*a^
K	2022	0.03 ± 0.00 ^B*a^	4.19 ± 0.046 ^B*a^	0.03 ± 0.00 ^A*a^	2.71 ± 0.05 ^B*b^	0.33 ± 0.01 ^A*ab^
K	2023	0.043 ± 0.006 ^A*a^	5.453 ± 0.493 ^A*a^	0.04 ± 0.01 ^A*a^	4.10 ± 0.435 ^A*a^	0.313 ± 0.023 ^A*a^
K	2024	0.027 ± 0.006 ^B*a^	3.87 ± 0.147 ^B*a^	0.023 ± 0.006 ^A*a^	2.63 ± 0.193 ^B*a^	0.33 ± 0.017 ^A*a^
K+EM-1	2022	0.03 ± 0.00 ^B*a^	4.53 ± 0.346 ^AB*a^	0.03 ± 0.00 ^B*a^	2.817 ± 0.025 ^B*ac^	0.32 ± 0.01 ^A*b^
K+EM-1	2023	0.043 ± 0.006 ^A*a^	5.727 ± 0.585 ^A*a^	0.04 ± 0.00 ^A*a^	3.757 ± 0.295 ^A*a^	0.303 ± 0.015 ^A*a^
K+EM-1	2024	0.023 ± 0.006 ^B*a^	4.003 ± 0.481 ^B*a^	0.027 ± 0.006 ^B*a^	2.843 ± 0.289 ^B*a^	0.33 ± 0.02 ^A*a^
K+GOIM	2022	0.03 ± 0.005 ^B*a^	4.555 ± 0.203 ^B*a^	0.037 ± 0.008 ^AB*a^	3.287 ± 0.308 ^B*a^	0.318 ± 0.019 ^B*b^
K+GOIM	2023	0.04 ± 0.00 ^A*a^	5.337 ± 0.242 ^A*a^	0.047 ± 0.006 ^A*a^	4.033 ± 0.155 ^A*a^	0.303 ± 0.006 ^B*a^
K+GOIM	2024	0.02 ± 0.00 ^C*a^	3.693 ± 0.076 ^C*a^	0.027 ± 0.006 ^B*a^	2.797 ± 0.042 ^B*a^	0.353 ± 0.006 ^A*a^
*p* values	T	0.62	0.23	0.08	0.14	0.05
*p* values	Y	<0.001	<0.001	<0.001	<0.001	<0.001
*p* values	T × Y	0.98	0.43	0.55	0.009	0.04

Means are presented ± SD (*n* = 3). Different letters denote statistically significant year effects (capital letters) and treatment effects (small letters). They begin in order, with letter (a/A) being the most significant. The star symbol (*) denotes the statistical interaction between two factors (year × fertilizer treatment). T: treatment; Y: year.

**Table 3:** Post hoc results of fertilizer effects on polyunsaturated fatty acid composition over years.

		C18:2	C18:3
Treatment	Year	Linoleic Acid	α-Linolenic Acid
CONTROL	2022	19.32 ± 0.306 ^B*bc^	0.03 ± 0.00 ^B*b^
CONTROL	2023	60.327 ± 3.827 ^A*a^	0.055 ± 0.005 ^A*a^
CONTROL	2024	6.333 ± 2.389 ^C*a^	0.03 ± 0.00 ^B*a^
EM-1	2022	32.075 ± 2.735 ^B*ab^	0.043 ± 0.006 ^A*a^
EM-1	2023	60.523 ± 4.308 ^A*a^	0.05 ± 0.00 ^A*a^
EM-1	2024	3.627 ± 1.342 ^C*a^	0.03 ± 0.00 ^B*a^
GOIM	2022	17.29 ± 0.262 ^B*d^	0.03 ± 0.00 ^B*b^
GOIM	2023	58.317 ± 1.25 ^A*a^	0.055 ± 0.005 ^A*a^
GOIM	2024	5.167 ± 1.677 ^C*a^	0.03 ± 0.00 ^B*a^
GOIM+EM-1	2022	10.683 ± 1.38 ^B*e^	0.04 ± 0.00 ^B*ab^
GOIM+EM-1	2023	59.93 ± 3.402 ^A*a^	0.06 ± 0.01 ^A*a^
GOIM+EM-1	2024	4.67 ± 1.333 ^C*a^	0.03 ± 0.00 ^B*a^
K	2022	14.29 ± 1.042 ^B*de^	0.037 ± 0.006 ^B*ab^
K	2023	60.27 ± 3.543 ^A*a^	0.06 ± 0.00 ^A*a^
K	2024	4.84 ± 1.569 ^C*a^	0.03 ± 0.00 ^B*a^
K+EM-1	2022	16.347 ± 0.897 ^B*cd^	0.04 ± 0.00 ^B*ab^
K+EM-1	2023	60.1967 ± 3.966 ^A*a^	0.055 ± 0.005 ^A*a^
K+EM-1	2024	9.343 ± 9.179 ^C*a^	0.03 ± 0.00 ^C*a^
K+GOIM	2022	31.978 ± 1.28 ^B*a^	0.045 ± 0.005 ^B*a^
K+GOIM	2023	59.297 ± 1.415 ^A*a^	0.06 ± 0.00 ^A*a^
K+GOIM	2024	4.027 ± 0.974 ^C*a^	0.03 ± 0.00 ^C*a^
*p* values	T	<0.001	0.002
*p* values	Y	<0.001	<0.001
*p* values	T × Y	<0.001	<0.001

Means are presented ± SD (*n* = 3). Different letters denote statistically significant year effects (capital letters) and treatment effects (small letters). They begin in order, with letter (a/A) being the most significant. The star symbol (*) denotes the statistical interaction between two factors (year × fertilizer treatment). T: treatment; Y: year.

**Table 4:** Post hoc results of fertilizer effects on saturated (SFAs), monounsaturated (MUFAs) and polyunsaturated (PUFAs) fatty acids over years.

Treatment	Year	SFA	MUFA	PUFA	PUFA ω-6	PUFA ω-3	MUFA/PUFA
CONTROL	2022	9.14 ± 0.13 ^B*bc^	71.18 ± 0.19 ^B*c^	19.68 ± 0.31 ^B*bc^	19.65 ± 0.33 ^B*b^	0.03 ± 0.00 ^B*cd^	3.62 ± 0.07 ^A*b^
CONTROL	2023	10.92 ± 0.28 ^A*a^	28.69 ± 3.65 ^C*a^	60.39 ± 3.83 ^A*a^	60.33 ± 3.83 ^A*a^	0.06 ± 0.01 ^A*a^	0.48 ± 0.09 ^B*a^
CONTROL	2024	8.93 ± 0.28 ^B*a^	84.67 ± 2.67 ^A*a^	6.40 ± 2.40 ^C*a^	6.33 ± 2.39 ^C*a^	0.06 ± 0.02 ^AB*a^	14.42 ± 4.79 ^AB*a^
EM-1	2022	9.72 ± 0.06 ^B*ab^	58.14 ± 2.65 ^B*d^	32.14 ± 2.73 ^B*a^	32.08 ± 2.74 ^B*a^	0.05 ± 0.01 ^A*a^	1.82 ± 0.23 ^A*c^
EM-1	2023	10.74 ± 0.09 ^A*a^	28.68 ± 4.287 ^C*a^	60.58 ± 4.319 ^A*a^	60.523 ± 4.308 ^A*a^	0.06 ± 0.01 ^A*a^	0.48 ± 0.102 ^B*a^
EM-1	2024	8.7 ± 0.106 ^C*a^	87.60 ± 1.31 ^A*a^	3.71 ± 1.34 ^C*a^	3.63 ± 1.34 ^C*a^	0.073 ± 0.058 ^A*a^	25.95 ± 9.85 ^AB*a^
GOIM	2022	9.25 ± 0.10 ^B*abc^	73.42 ± 0.18 ^B*bc^	17.33 ± 0.27 ^B*bc^	17.29 ± 0.26 ^B*bc^	0.03 ± 0.00 ^C*c^	4.24 ± 0.07 ^A*a^
GOIM	2023	11.03 ± 0.31 ^A*a^	30.59 ± 1.45 ^C*a^	58.37 ± 1.26 ^A*a^	58.32 ± 1.25 ^A*a^	0.06 ± 0.01 ^A*a^	0.53 ± 0.04 ^B*a^
GOIM	2024	9.19 ± 0.32 ^B*a^	85.60 ± 1.38 ^A*a^	5.21 ± 1.68 ^C*a^	5.17 ± 1.68 ^C*a^	0.04 ± 0.01 ^B*a^	18.04 ± 7.47 ^AB*a^
GOIM+EM-1	2022	9.24 ± 0.377 ^B*abc^	80.04 ± 1.75 ^B*a^	10.72 ± 1.38 ^B*d^	10.68 ± 1.38 ^B*d^	0.04 ± 0.00 ^B*b^	7.56 ± 1.08 ^A*ab^
GOIM+EM-1	2023	10.8 ± 0.20 ^A*a^	29.21 ± 3.21 ^C*a^	59.99 ± 3.41 ^A*a^	59.93 ± 3.40 ^A*a^	0.06 ± 0.01 ^A*a^	0.49 ± 0.08 ^B*a^
GOIM+EM-1	2024	8.64 ± 0.32 ^B*a^	86.65 ± 1.58 ^A*a^	4.71 ± 1.33 ^C*a^	4.67 ± 1.33 ^C*a^	0.04 ± 0.00 ^B*a^	19.36 ± 5.13 ^A*a^
K	2022	9.02 ± 0.16 ^B*c^	76.65 ± 0.89 ^B*ab^	14.33 ± 1.05 ^B*cd^	14.29 ± 1.04 ^B*cd^	0.04 ± 0.01 ^B*bc^	5.37 ± 0.47 ^A*ab^
K	2023	11.27 ± 0.21 ^A*a^	28.40 ± 3.56 ^C*a^	60.33 ± 3.55 ^A*a^	60.27 ± 3.54 ^A*a^	0.06 ± 0.01 ^A*a^	0.47 ± 0.09 ^B*a^
K	2024	8.65 ± 0.19 ^B*a^	86.41 ± 1.38 ^A*a^	4.94 ± 1.53 ^C*a^	4.84 ± 1.57 ^C*a^	0.097 ± 0.090 ^AB*a^	18.58 ± 5.18 ^A*a^
K+EM-1	2022	9.27 ± 0.30 ^B*abc^	74.35 ± 1.10 ^B*bc^	16.39 ± 0.90 ^B*bc^	16.35 ± 0.90 ^B*bc^	0.04 ± 0.00 ^B*b^	4.55 ± 0.33 ^A*ab^
K+EM-1	2023	11.18 ± 0.27 ^A*a^	28.56 ± 4.23 ^C*a^	60.26 ± 3.97 ^A*a^	60.20 ± 3.97 ^A*a^	0.06 ± 0.01 ^A*a^	0.48 ± 0.1 ^B*a^
K+EM-1	2024	8.91 ± 0.65 ^B*a^	81.71 ± 9.79 ^A*a^	9.38 ± 9.18 ^C*a^	9.34 ± 9.18 ^C*a^	0.04 ± 0.00 ^B*a^	18.53 ± 17.57 ^AB*a^
K+GOIM	2022	9.78 ± 0.15 ^B*a^	58.19 ± 1.14 ^B*d^	32.03 ± 1.29 ^B*a^	31.98 ± 1.28 ^B*a^	0.05 ± 0.01 ^B*a^	1.82 ± 0.11 ^B*c^
K+GOIM	2023	11.05 ± 0.14 ^A*a^	29.59 ± 1.53 ^C*a^	59.36 ± 1.42 ^A*a^	59.30 ± 1.42 ^A*a^	0.06 ± 0.00 ^A*a^	0.50 ± 0.04 ^C*a^
K+GOIM	2024	8.76 ± 0.11 ^C*a^	87.17 ± 1.06 ^A*a^	4.07 ± 0.97 ^C*a^	4.03 ± 0.97 ^C*a^	0.04 ± 0.00 ^C*a^	22.25 ± 5.13 ^A*a^
*p* values	T	0.18	<0.001	<0.001	<0.001	0.02	0.01
*p* values	Y	<0.001	<0.001	<0.001	<0.001	<0.001	<0.001
*p* values	T × Y	0.008	<0.001	<0.001	<0.001	0.03	0.008

Means are presented ± SD (*n* = 3). Different letters denote statistically significant year effects (capital letters) and treatment effects (small letters). They begin in order, with letter (a/A) being the most significant. The star symbol (*) denotes the statistical interaction between two factors (year × fertilizer treatment). T: treatment; Y: year.

**Table 5:** Main climatic data recorded in the experimental area from 2022 to 2024.

Year	Month	Average Temperature °C	Average Humidity (%)	Rainfall (mm)
2022	May	17.6	61.86	29.5
2022	June	22.5	58.12	41.1
2022	July	23.7	48.58	25.0
2022	August	23.8	61.14	29.5
2022	September	15.1	76.81	84.6
2023	May	15.92	73.21	78.3
2023	June	19.77	69.81	48.6
2023	July	22.82	66.44	29.0
2023	August	22.26	71.26	113.7
2023	September	19.92	69.41	6.3
2024	May	17.41	67.56	37.7
2024	June	21.47	71.83	65.1
2024	July	24.72	61.84	23.1
2024	August	24.59	62.46	33.3
2024	September	17.83	68.42	82.8

**Table 6:** Layout of the experimental site (randomized complete block design, three replications).

Replication I	Replication II	Replication III
CONTROL	K+EM-1	GOIM
POTASSIUM K	GOIM	GOIM+EM-1
GOIM	EM-1	K+GOIM
EM-1	POTASSIUM K	CONTROL
K+GOIM	CONTROL	K+EM-1
K+EM-1	GOIM+EM-1	EM-1
GOIM+EM-1	K+GOIM	POTASSIUM K

## Data Availability

The data presented in this study are available on request from the corresponding author.
